# Enhanced resection of exophytic gastric gastrointestinal stromal tumor using traction-assisted subserosal dissection and endoscopic suturing

**DOI:** 10.1055/a-2722-9685

**Published:** 2025-11-05

**Authors:** Chen-Shuan Chung, Chen-Huan Yu

**Affiliations:** 1 46608 Division of Gastroenterology and Hepatology, Department of Internal Medicine, Far Eastern Memorial Hospital, New Taipei City, Taiwan; 2 Taiwan Association for the Study of Intestinal Diseases (TASID), Taoyuan City, Taiwan; 3 34895 Program A, Department of Electrical Engineering, Yuan Ze University, Taoyuan, Taiwan

A 49-year-old man was incidentally found to have a gastric subepithelial lesion located at the lesser curvature of the upper body during routine endoscopic screening ( Fig. 1 ). Although the lesion appeared small on conventional endoscopy, endoscopic ultrasound (EUS) revealed an exophytic, hypoechoic mass with heterogeneous echotexture and internal hyperechoic foci, originating from the muscularis propria layer. During a 6-month surveillance period, the lesion increased from 1.2 to 1.5 cm. Contrast-enhanced harmonic EUS (CEH-EUS) with intravenous Sonazoid revealed early and heterogeneous enhancement ( Fig. 2 ). Due to high-risk EUS features and patient preference, endoscopic subserosal dissection (ESSD) was undertaken ( Video 1 ).

**Fig. 1:**
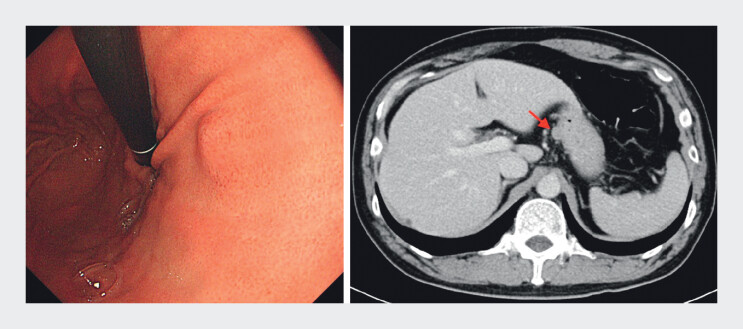
An endoscopically visualized small but exophytic tumor was identified at the lesser curvature of the upper gastric body.

**Fig. 2:**
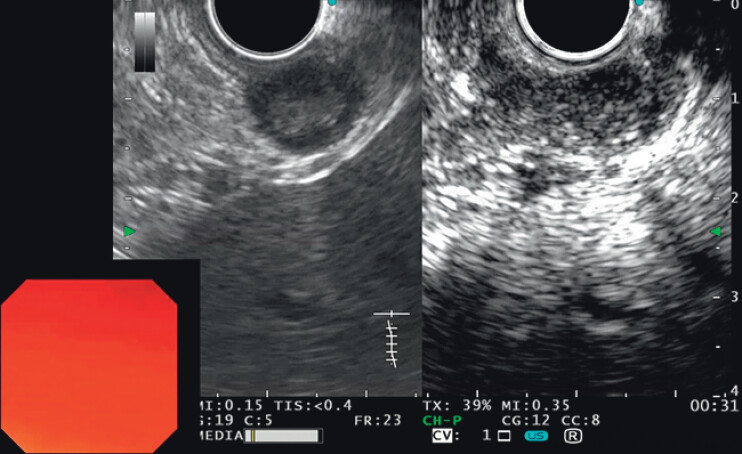
EUS showed internal hyperechoic foci; CEH-EUS revealed early and heterogeneous enhancement. EUS, endoscopic ultrasound; CEH-EUS, Contrast-enhanced harmonic EUS.

Traction-assisted endoscopic subserosal dissection and endoscopic suturing of an exophytic gastric GIST.Video 1

The procedure was performed using a GIF-H290Z gastroscope (Olympus, Japan) with the EVIS X1 system (Olympus, Japan) and an electrosurgical knife (DualKnife J; Olympus, Japan). Following submucosal injection of a glycerol solution mixed with indigo carmine, a partial mucosal incision was made on the anal side using EndoCut I settings (effect 2, duration 3, interval 2; VIO300D, ERBE, Germany). To facilitate subserosal exposure, the clip-and-snare traction technique was employed, enabling effective visualization of exophytic tumor capsule for further dissection ( Fig. 3 ). ESSD was completed without perforation and the lesion was resected en bloc. Closure of the mucosal defect was achieved with an endoscopic suturing system (OverStitch NXT; Boston Scientific, USA; Fig. 4 ). The patient tolerated the procedure well and resumed oral intake on postoperative day 1 with discharged uneventfully.

**Fig. 3:**
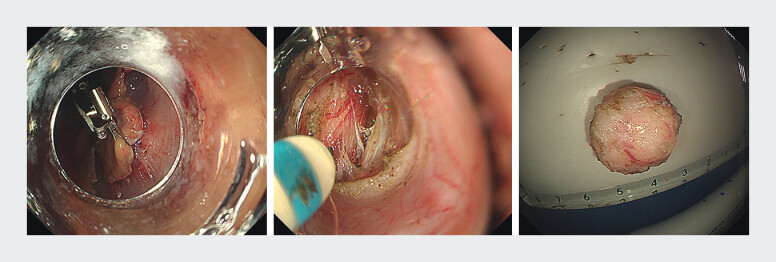
Clip-and-snare traction exposing the dissection plane, facilitating en bloc resection.

**Fig. 4:**
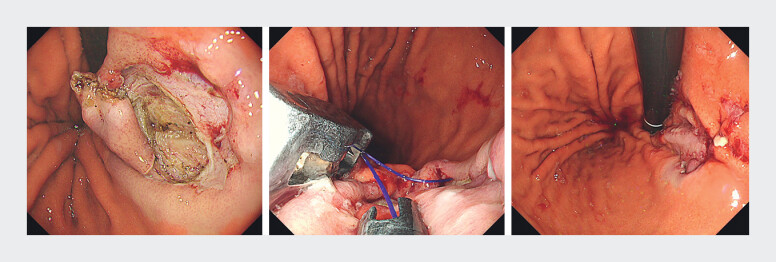
Endoscopic closure of the mucosal defect using the OverStitch NXT suturing system.

This case demonstrates the feasibility of ESSD for exophytic gastric GISTs using traction and endoscopic suturing. The clip-and-snare technique provided excellent exposure of the subserosal plane and tumor capsule 1 , while OverStitch NXT ensured secure full-thickness closure of the mucosal defect 2 . CEH-EUS aided in the differential diagnosis and supported the decision for endoscopic resection 3 . With careful patient selection, ESSD may offer a minimally invasive, organ-preserving alternative to surgery for exophytic gastric GISTs 4 .

Endoscopy_UCTN_Code_TTT_1AO_2AG_3AF

## Notes

Endoscopy E-Videos https://eref.thieme.de/e-videos
*E-Videos* is an open access online section of the journal *Endoscopy* , reporting on interesting cases and new techniques in gastroenterological endoscopy. All papers include a high-quality video and are published with a Creative Commons CC-BY license. Endoscopy E-Videos qualify for HINARI discounts and waivers and eligibility is automatically checked during the submission process. We grant 100% waivers to articles whose corresponding authors are based in Group A countries and 50% waivers to those who are based in Group B countries as classified by Research4Life (see: https://www.research4life.org/access/eligibility/ ). This section has its own submission website at https://mc.manuscriptcentral.com/e-videos .
